# Effects of Mind–Body Movements on Balance Function in Stroke Survivors: A Meta-Analysis of Randomized Controlled Trials

**DOI:** 10.3390/ijerph15061292

**Published:** 2018-06-20

**Authors:** Liye Zou, Albert Yeung, Chunxiao Li, Shin-Yi Chiou, Nan Zeng, Huey-Ming Tzeng, Lin Wang, Zhanbing Ren, Taquesha Dean, Garrett Anthony Thomas

**Affiliations:** 1 Department of Physical Education, Wuhan University of Technology, Wuhan 430070, China; 2 Department of Sports Science and Physical Education, the Chinese University of Hong Kong, Shatin, Hong Kong, China; 3 Depression Clinical and Research Program, Harvard Medical School, Harvard University, Boston, MA 02114, USA; AYEUNG@mgh.harvard.edu (A.Y.); TDEAN4@mgh.harvard.edu (T.D.); GTHOMAS12@mgh.harvard.edu (G.A.T.); 4 Department of Health and Physical Education, The Education University of Hong Kong, Tai Po, Hong Kong, China; cxli@eduhk.hk; 5 School of Sport, Exercise and Rehabilitation Sciences, University of Birmingham, Birmingham B15 2TT, UK; s.chiou12@imperial.ac.uk; 6 School of Kinesiology, University of Minnesota-Twin Cities, Minneapolis, MN 55455, USA; zengx185@umn.edu; 7 College of Nursing, University of Saskatchewan, 104 Clinic Place, Saskatoon, SK S7N 2Z4, Canada; tzenghm@gmail.com; 8 Department of Physical Education, Shenzhen University, Shenzhen 518060, China; rzb@szu.edu.cn

**Keywords:** Tai Chi, Yoga, mindfulness movement, stroke, rehabilitation

## Abstract

*Objective*: We performed a systematic review with meta-analysis and meta-regression to determine if mind–body movements (MBM) could be effective in rehabilitating balance function among stroke survivors. *Methods*: A literature search was conducted using major Chinese and English electronic databases from an inception until January 2018. Randomized controlled studies were included in our meta-analysis. Data was independently extracted by two review authors using a pre-developed table and confirmed by a third party to reach a consensus. Pooled effect size (Hedge’s *g*) was computed while the random-effect model was set. *Results*: The meta-analytic results showed a significant benefit of the MBM intervention on increased balance function compared to the control groups (Hedge’s *g* = 1.59, *CI* 0.98 to 2.19, *p* < 0.001, *I*^2^ = 94.95%). Additionally, the meta-regression indicated that the total number of sessions (β = 0.00142, 95% *CI* 0.0039 to 0.0244, *p* = 0.0067) and dose of weekly training (β = 0.00776, 95% *CI* 0.00579 to 0.00972, *p* = 0.00) had significantly positive effects on balance function. *Conclusions*: The study encouraging findings indicate the rehabilitative effect of a MBM intervention for balance function in stroke survivors. However, there were significant limitations in the design among several of the included trials. Additional studies with more robust methodologies are needed to provide a more definitive conclusion.

## 1. Introduction

Strokes are the second-leading cause of death worldwide and are considered one of the most common causes of long-term disabilities [1]. According to the World Health Organization (WHO), approximately15 million individuals worldwide experience a first-ever stroke each year [2]. Of these, there are an estimated five million stroke survivors who are left permanently disabled [2], placing a burden on family and society. The social-economic costs (such as medications, hospitalization, job loss/reduced productivity) of Chinese stroke survivors were $16.08 billion in 2008 and this number continues to increase [3]. Furthermore, approximately 80% of the stroke survivors suffer from balance disorders due to visually guided performance deficit, loss of proprioception, and altered walking patterns [4,5]. Compared to healthy age-matched adults, stroke survivors with impaired balance are seven times more likely to fall, which is associated with a higher risk of fractures, ill-being, anxiety, depression, and even mortality [6,7].

According to the Centers for Disease Control and Prevention, 67% of the stroke survivors require treatments to improve their balance [8,9]. To help them attain the best possible quality of life, physical therapy is commonly prescribed as the main approach to pharmacotherapy to help stroke survivors regain motor function and the ability to control their balance [10,11]. However, using physical therapy to improve balance requires time-consuming input from therapists while they carry out the interventions. In addition, it is usually limited to authorized rehabilitation centers which are not always affordable or accessible by stroke survivors [12]. Therefore, it is crucial to identify complementary approaches for balance rehabilitation for stroke survivors that are low cost and community-based, with the goal of maximizing the recovery of balance function post-stroke.

Mind–body movements (e.g., Tai Chi, yoga or Qigong) may be a complementary rehabilitation method for balance rehabilitation in stroke survivors [13,14,15], since they share similar mechanisms of movement that align with conventional rehabilitation methods, such as the Bobath technique (focusing on quality of movement and correct movement patterns to strengthen kinesthetic awareness, leading to better postural stability) and proprioceptive neuromuscular facilitation [16]. Smooth and well-coordinated whole body movements in these mind–body movements (MBM) routines assist with the control of voluntary weight-shifting, which could potentially strengthen the core and lower limb muscles of stroke survivors, therefore naturally leading to better balance performance [17,18]. Practicing MBM also requires musculoskeletal relaxation when integrated with breath control and mental focus, which could lead to decreased fatigue and increased patient adherence to achieve faster recovery [19,20].

Recently, a number of empirical studies have been conducted that investigate the rehabilitative effects of MBM on balance within stroke survivors [21,22,23,24,25,26,27]. Subsequently, review studies have emerged on this topic; however, based on qualitative analyses, all of the reviews have only paid attention to Tai Chi or yoga [28,29,30,31]. Furthermore, most of the reviews do not include Chinese academic databases due to the language barrier. Moreover, there have been increasing data from the emerging literature showing that Tai Chi and Qigong have rehabilitative effects on balance function in stroke survivors. Therefore, a meta-analytic systematic review is needed to critically assess available evidence concerning the effects of these three MBM on balance function in stroke survivors. We expect that the updated findings of this review could allow scholars and health professionals to design and develop effective MBM rehabilitative protocols that can speed up the balance recovery process and maximize treatment effects.

## 2. Methods

### 2.1. Protocol and Registration

This review was carried out in accordance with the Preferred Reporting Items for Systematic Review and Meta-Analysis (PRISMA) guidelines and the checklist was completed [32]. The project was registered with the International Prospective Register of Systematic Reviews (No. CRD4201808 5213) to eliminate duplicates.

### 2.2. Information Sources and Search

The leading reviewer (L.Z.) performed the literature search (January 2018) using both English (e.g., PubMed, Physiotherapy Evidence Database, Cochrane Library, and WHO International Clinical Trials Registry Platform) and Chinese (e.g., China National Knowledge Infrastructure, Wanfang, and Chinese Clinical Trial Registry) electronic databases without restricting publication dates. Two groups of search terms were used in a combined manner: (1) “stroke”, “cerebrovascular accident”, “brain attack”, “brain ischemia”, or “intracranial hemorrhage”; and (2) “Yoga”, “Tai Chi/Taiji”, or “Qigong”. Relevant studies were also manually identified based on cross-referencing searches.

### 2.3. Eligibility Criteria and Study Selection

Studies that met the following criteria were included: (1) was a randomized controlled trial (RCT); (2) were published in peer-reviewed Chinese/English journals; (3) given that yoga, Tai Chi, and Qigong are ranked as the top 3 complementary therapies according the National Health Interview Survey [33], usage of at least one of these three MBM as the primary interventions for at least 2 weeks; (4) samples of at least 15 stroke survivors; and (5) reporting of quantitative data for computing the standardized training effect (Hedge’s *g*) on balance function. Controlled trials without randomization, cross-sectional studies, case reports/series, and review studies were excluded. Two researchers (L.Z. and N.Z.) independently screened the title and abstract and the full text was retrieved when necessary. If there was disagreement between the two researchers, a third reviewer (A.Y.) was involved in the discussion until a consensus was reached.

### 2.4. Data Items and Collection Processes

Researchers (L.Z. and N.Z.) used a pre-developed table (Table 1) for extracting data items which included references (i.e., name of the leading author, year of publication, and study location), participant characteristics (initial sample size and attribution rate, mean age/age range, course of disease, and stroke type, i.e., ischemic or hemorrhage), intervention components (types, training frequency and length, mode of combination, training mode, weekly training dosage of at least 150 min weekly, which, per the National Physical Activity Guidelines, is recommended for persons with physical disabilities) [34], total number of training sessions, control group activity (treatment mode), and the balance/instrument used. Two researchers (L.Z. and N.Z.) independently extracted data from the eligible studies. The data entry was then cross-checked by a third party (A.Y.).

### 2.5. Risk of Bias in Individual Studies

Based on previous studies [35,36], we adapted the Physiotherapy Evidence Database (PEDro) scale to assess risk of bias in the eligible studies. Given that the blinding of participants and instructor(s) are impractical in non-pharmacological interventions, we removed the two items that assessed that. The updated PEDro scale consisted of nine items: randomization, concealed allocation, similar baseline, blinding of assessors, more than 85% retention, missing data management/intention-to-treat analysis, between-group comparison, point measure of variability, and co-intervention, which should be either avoided in the trial design or similar between the index and control groups. Points were only awarded if authors explicitly reported the information. Each study could have earned a maximum of nine points, with higher scores indicating lower risk of bias.

### 2.6. Synthesis of Results, Risk of Bias across Studies, and Additional Analysis

Effect sizes (Hedge’s *g*) across individual studies were pooled using the random-effect model in a Comprehensive Meta-Analysis Software. The value of I-squared was used to determine the heterogeneity of the effect sizes across the RCTs selected: (1) small = 25%; (2) medium = 50%; (3) and large = 75%. A series of sub-group analyses were conducted and analyzed, including intervention duration (<12 weeks vs. ≥12 weeks), training frequency (<5 sessions/week vs. ≥5 sessions/week), session length (<45 min vs. ≥45 min, which according to General Guidelines for Exercise Training and Progression for Stroke Recovery and Rehabilitation, 45 min of exercise training is tolerable) [37], mode of combination (separate vs. simultaneous), weekly training duration of at least 150 min (yes vs. no), and total sample size (less than 60 vs. 60+) were conducted. A meta-regression analysis was conducted with the total number of training sessions and dose of weekly training as the predictors. Finally, we used the funnel plot and Egger’s regression intercept test to determine whether publication bias existed.

## 3. Results

### 3.1. Study Selection

**Figure 1:**
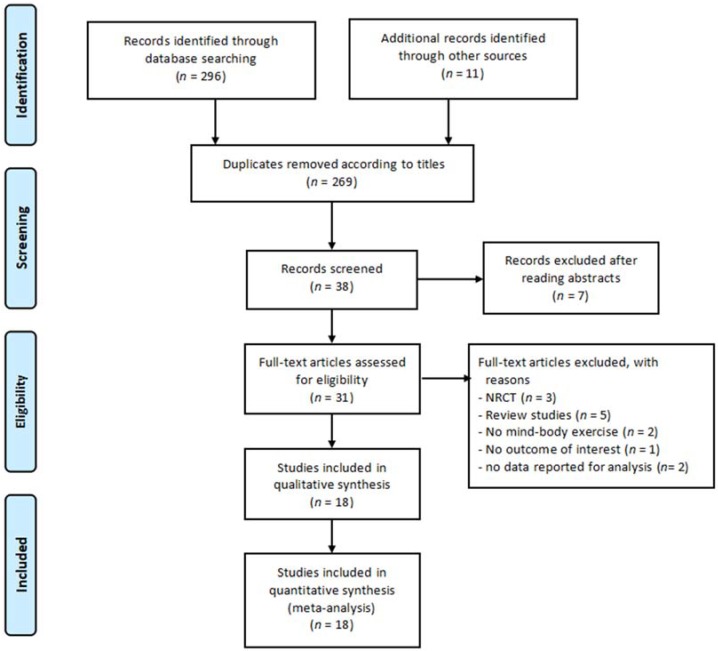
Procedures of study selection. (Note: NRCT means non-randomized control trial.)

Figure 1 shows the procedures of study selection. The electronic and manual searches resulted in 307 articles. Based on the titles of initially identified articles, there were 269 duplicates and therefore they were removed. After removing the 269 duplicates, 38 studies remained for screening. After reading abstract of the remaining 38 articles, seven irrelevant records were excluded. Based on the eligibility criteria, 13 studies were removed because they were either not RCTs (*n* = 3), were review studies (*n* = 5), had no MBM intervention (*n* = 2), provided no outcome of interest (*n* = 1), or had no data reported for analysis (*n* = 1).This left only 18 eligible RCTs to be included for the meta-analysis.

### 3.2. Study Characteristics

The characteristics of the 18 included studies are presented in Table 1. These studies were published between 2009 and 2017. Most of them were conducted in mainland China, followed by the USA (*n* = 2), Austria (*n* = 1), Japan (*n* = 1), and South Korea (*n* = 1). The total number of participants was 1189 (MBM = 594 and Control = 595), and the study’s sample sizes varied from 22 to 224, with attrition rates ranging from 1.3% to 21%. The mean age of participants varied from 42.8 to 77.59 years old (range between 33 and 82 years). Both acute and chronic strokes were included and the average duration of disease was between 14.5 and 47.34 days, 6.22 and 7.01 weeks, or 1 to 81.6 months. The number of patients with ischemic (*n* = 415) and hemorrhage strokes (*n* = 231) were also reported in some RCTs. The intervention duration ranged from 4 to 12 weeks, with 1 to 7 sessions per week, each lasting 15 to 90 min per session. Only 11 studies reported weekly training sessions that lasted more than 150 min. The total number of training sessions ranged between 8 and 84 sessions, with a total weekly training dose of 90 to 330 min. Tai Chi was the most frequently used intervention (*n* = 12), followed by Qigong (*n* = 4), and yoga (*n* = 2). In 77.78% of the studies selected, the MBM intervention was combined with either balance training, educational programming, Bobath techniques and drug therapy, general rehabilitation and physical therapy, device-assisted rehabilitation, Prokin balance feedback training, or the usual treatment routine. The three different training modes were individual practice, group-based practice, and mixed mode. Adverse events, such as death, hospitalizations, falls, or injuries did not occur during MBM intervention.

### 3.3. Risk of Bias within Studies

Table 2 summarizes the results of the methodological quality assessment. The summed scores of the PEDro scale across the eligible studies were relatively high, ranging from 5 to 9. Specifically, 13 studies fell between the range of 7–9 points, followed by 4 studies with 6 points, and one study with 5 points. Roughly 77.78% of the 18 RCTs selected did not clearly report use of concealed allocation and blinded assessors. The additional points that were deducted were due to high attribution rates [31], missing data management [29,30,32,43,46], between-group comparisons [28], and co-intervention equivalence [47].

### 3.4. Effects of MBM on Improvement of Balance

**Figure 2:**
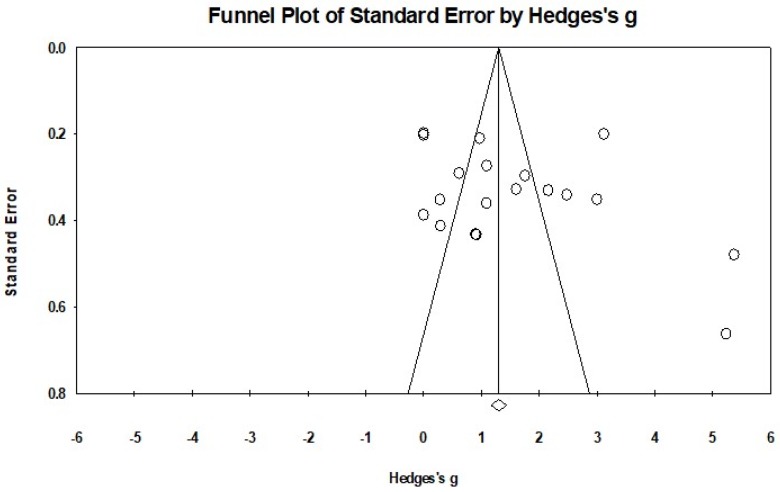
The Funnel Plot of included dependent effect sizes (*k* = 18).

**Figure 3:**
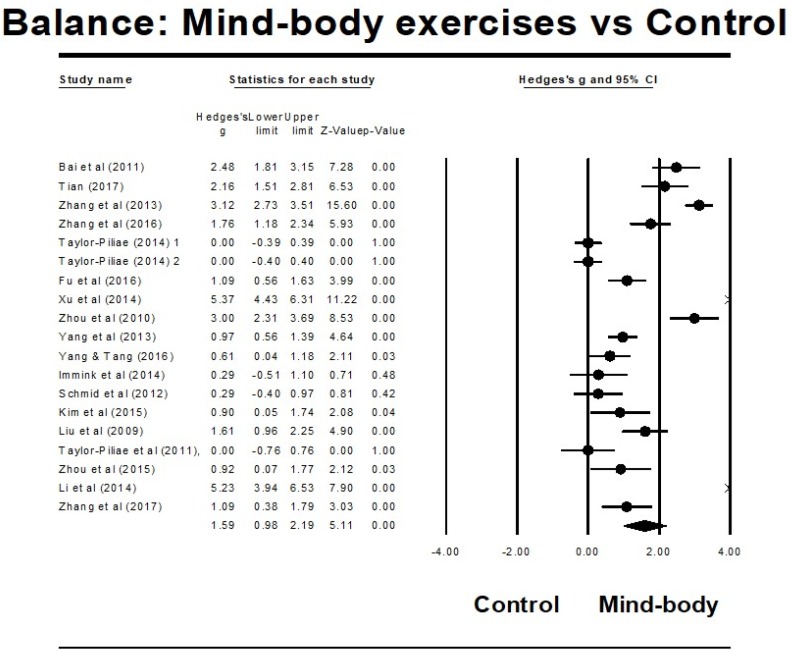
Effect of mindfulness-based movement intervention on balance function.

The Egger’s regression test was not significant (Egger’s regression intercept = 4.679, *p* = 0.172), along with a relatively symmetrical Funnel plot (Figure 2). The included studies used three different instruments (the Berg Balance Scale, the functional Reach Test, and the Timed Balance Test) to examine the effects of the MB exercise intervention on balance function in comparison to a control group. A higher positive value for all the balance tests indicated better balance performance. The aggregated results demonstrated a significant benefit of MBM interventions on balance function: (Hedge’s *g* = 1.59, *CI* 0.98 to 2.19, *p* < 0.001, *I*^2^ = 94.95%; Figure 3).

### 3.5. Moderator Analysis

The results of the sub-group analyses are displayed in Table 3. There were significant effects for training frequency (Q[1] = 4.69, *p* = 0.03), session length (Q[1] = 14.21, *p* = 0.00), and mode of combination (Q[1] = 7.59, *p* = 0.01) on balance function. On the other hand, no significant effects were found for intervention duration (Q[1] = 0.07, *p* = 0.79), weekly training duration (Q[1] = 0.88, *p* = 0.35), or sample size (Q[1] = 0.91, *p* = 0.34). In addition, the results of the meta-regression indicated that the total number of sessions (β = 0.00142, 95% CI 0.0039 to 0.0244, *p* = 0.0067) and dose of weekly training (β = 0.00776, 95% CI 0.00579 to 0.00972, *p* = 0.00) had significantly positive effects on balance function.

## 4. Discussion

Our review critically evaluated and statistically synthesized available evidence on the effects of MBM (Tai Chi, Qigong and yoga) on balance function among stroke survivors. Based on the evidence available from previous literature, our review suggests that implementing these exercises may improve balance function among stroke survivors. Moreover, data from the emerging literature has increasingly highlighted the efficacy of augmentative Tai Chi or Qigong training for patients recovering from the acute phase of a stroke. Given previous concerns regarding the efficacy and safety of yoga as a stroke rehabilitation treatment due to a lack of information, these findings are encouraging [49]. To our knowledge, this is the first meta-analysis that has examined the effects of MBM interventions on post-stroke rehabilitation. The findings from this study are also significant from a public health perspective given that many stroke survivors experience varying degrees of balance loss, which then affects their mobility, physical functioning, quality of life, mood and independence [50]. Given these findings, MBM may be implemented as a complementary treatment to stroke patients as a safe and inexpensive option to aid in achieving more favorable outcomes.

Presently, the exact mechanisms through which Tai Chi, Qigong and yoga affect balance in stroke survivors are unknown. However, one modern theory suggests that these MBM elicit positive changes by enhancing physiological proprioception—specifically by pairing posture, movement and breath control practices with a special state of awareness. This enhanced state of awareness thereby improves and strengthens the overall state of vegetative regulation, also known as homeostasis, which may mediate the positive outcomes associated with these MBM [51].

Overall, there are many advantages of employing MBM as an add-on therapy for post-stroke patients. It is easy to learn, has few known side effects and is widely accessible to people of all ages and physical strength. In addition, these MBM facilitate relaxation and personal integration aspects (e.g., an enhanced ability to sustain attention [52]), which contribute to increased mindful awareness and personal acceptance [53]. These integration methods may be beneficial for patients and may ultimately transcend post-stroke treatment and elicit positive outcomes in other areas of the patients’ lives, as well. However, some disadvantages are that qualified Tai Chi, Qigong and yoga instructors may not be available in some areas thus limiting accessibility. Furthermore, long-term adherence of these practices tends to be low which may reduce the overall efficacy and feasibility of these practices.

One major strength of this review is that it included published empirical studies in both English and Chinese. This expansive inclusion is appropriate and important since most of the current studies assessing the rehabilitative effects of MBM for balance of stroke patients were conducted in China and were published mostly in the Chinese language. Therefore, by including articles in Chinese, the findings from researchers published in Chinese peer-reviewed journals are included and acknowledged. Other strengths of this meta-analysis include: the use of a standardized scale to assess the methodological quality of the studies, the use of a recognized meta-analytic method to evaluate the magnitude of the MB exercises’ intervention effect (pooled effect size), the variations in the frequency and duration of meditative movements practice (moderator analyses), and the extent of symmetry of the effect sizes (funnel plot and Egger’s regression) on balance function.

This review is not without limitations even if encouraging findings are found. First, Tai Chi, Qigong and yoga in this review are formulated into MBM and their quantitative data on balance are synthesized. While key researchers in MB research propose that Tai Chi, Qigong and yoga can all be viewed as meditative movements given that they use similar techniques [54], further studies are needed to discern whether there are mechanistic differences between these practices and confirm that these exercises have comparable effects for post-stroke rehabilitation. Secondly, many of the studies used non-blinded assessors, so a subjectivity and social desirability bias may influence the interpretation of these research findings. Third, most of the studies did not offer MBM as a monotherapy, but as an alternative treatment to the interventions already received by the patients. Therefore, it may be difficult to conclude whether the positive outcomes were attributed to stand-alone MBM, a synergetic intervention effect, or the treatment already being received by the patients. Nonetheless, our data provides support for MBM as an alternative treatment for stroke survivors to improve their balance function. Another limitation was that, across all studies, numerous different interventions were received by the control groups which made the interpretations and comparisons of outcomes difficult. Additionally, the frequency and the duration of the MBM practices varied extensively between the different studies which further complicated our analyses. For example, in the moderator analyses, while higher training frequency, mode of combination, session length, weekly training dosage, and total number of sessions were associated with better outcomes, a longer intervention program (≥12 weeks) and longer weekly training intervention (≥150 min) were not. Such findings are hard to make specific recommendations regarding the dosage of the intervention. Therefore, future studies should be conducted to further establish effective parameters for treatment and define the critical point at which the duration of treatment becomes ineffective. Other limitations included the fact that most of the studies were conducted in Asia, therefore it remains unclear whether the findings are generalizable to non-Asian populations. Interestingly, the four studies that were performed in Western countries all showed negative outcomes, while the remaining studies conducted in non-Western countries showed positive outcomes [27,28,29,30]. Therefore, these findings raise the question of generalizability and whether the interventions could be comparable between different regions. Lastly, given that studies reporting positive or significant results are more likely to be published and outcomes are that are statistically significant have greater likelihood of being reported, this may lead to publication bias.

## 5. Conclusions

The findings of this study suggest that Tai Chi, Qigong or yoga could be used as an alternative treatment for post-stroke patients to improve balance function. There were significant weaknesses in the design of the analyzed studies and the outcomes varied in different regions. Future studies with more robust methodology will be needed to provide a more definitive conclusion.

## Figures and Tables

**Table 1:** Characteristics of studies selected.

Reference (Country)	Participant Characteristics				Mind–Body Intervention					Control Group Activity	Outcomes (Instrument)
	ISZ (AT)	Mean Age or Age Range (yr)	Course of Disease	Ischemic/Hemorrhage	Training Frequency and Length (MB Component)	Mode of Combination	Training Mode	(Training Dose) ≥150-min weekly	No. of Sessions (Total Volume)		
Taylor-Piliae [27], USA	145 (10%)				60 min × 3 sessions/wk, 12 wks (24-style Tai Chi)	Separate	Group	(180 min) Yes	36 (2160 min)	CG1: strength and range of movement exercises; CG2: phone call	Balance (TBT)
	MB: 53	MB: 71.5 (10.3)	MB: 39 (40.2) M	MB: 33/12							
	CG1: 44	CG1: 69.6 (9.4)	CG1: 33 (58.7) M	CG1: 32/8							
	CG2: 48	CG2: 68.2 (10.3)	CG2: 38.7 (46.7) M	CG2: 30/14							
				16 unknown							
Taylor-Piliae et al. [28], China	28 (11%)				60 min × 3 sessions/wk, 12 wks (24-style Tai Chi)	Separate	Group	(180 min) Yes	36 (2160 min)	Usual treatment	Balance (TBT)
	MB: 16	MB: 72.8 (10.1)	MB: 58.3 (46.7) M	MB: 12/4							
	CG: 12	CG: 64.5 (10.9)	CG: 47.9 (42.5) M	CG: 9/3							
Schmid et al. [29], USA	47 (9%)				60 min × 2 sessions/wk, 8 wks (Yoga)	Simultaneous (Usual treatment)	Group	(120 min) No	16 (960 min)	Usual treatment	Balance (BBS)
	MB: 37	MB: 63.9 (8.7)	MB: 54.9 (43.2) M	MB: 26/NR							
	CG: 10	CG: 60.2 (8.9)	CG: 36.4 (23.6) M	CG: 5/NR							
Immink et al. [30], Austria	25 (12%)				90 min × 1 session/wk (group), 10 wks + 40 min × 6 sessions (individual) (Yoga)	Simultaneous (Usual treatment)	Mixed	(330 min) Yes	70 (9100 min)	No treatment	Balance (BBS)
	MB: 12	MB: 56.1 (13.6)	MB: 81.6 (77.5) M	NR							
	CG: 13	CG: 63.2 (17.4)	CG: 23.3 (12.5) M								
Yang & Tang [31], China	62 (21%)				40 min × 3 sessions/wk, 8 wks (Tai Chi)	Simultaneous (general rehabilitation)	Group	(120 min) No	24 (960 min)	General rehabilitation	Balance (BBS)
	MB: 32	MB: 51.43 (15.63)	MB: 42.26 (19.83) d	MB: 20/8							
	CG: 30	CG: 54.85 (11.85)	CG: 40.57 (23.12) d	CG: 16/5							
Kim et al. [32], Korea	24 (8%)				60 min × 2 sessions/wk, 6 wks (simplified Tai Chi)	Simultaneous (general rehabilitation + physical therapy)	Group	(120 min) No	12 (720)	General rehabilitation + physical therapy	Dynamic balance (FRT)
	MB: 12	MB: 53.45 (11.54)	NR	NR							
	CG: 12	CG: 55.18 (10.2)									
Zhou, Li et al. [33], China	22 (0%)	35–70	<6 M	NR	60 min × 5 sessions/wk, 4 wks (Tai Chi exercise)	Simultaneous (device-assisted rehabilitation)	NR	(300 min) Yes	20 (1200 min)	Device-assisted rehabilitation	Balance (BBS)
	MB: 11										
	CG: 11										
Yang et al. [38], China	100 (0%)			Unable to identify	45 min × 6 sessions/wk, 4 wks (Tai Chi)	Separate	NR	(270 min) Yes	24 (1080 min)	Exercise rehabilitation	Balance (BBS)
	MB: 50	MB: 54.3 (13.8)	MB: 44.7 (18.4) d								
	CG: 50	CG: 55.2 (14.6)	CG: 42.6 (16.7) d								
Zhang et al. [39], China	34 (0%)		<6 M	NR	60 min × 5 sessions/wk, 4 wks (Tai Chi)	Separate	Group	(300 min) Yes	20 (1200 min)	General rehabilitation	Balance (BBS)
	MB: 17	MB: 43.5 (4.7)									
	CG: 17	CG: 42.8 (5.1)									
Fu et al. [40], China	60 (0%)		<3 M		15 min × 6 sessions/wk, 8 wks (24-style Tai Chi)	Simultaneous (General rehabilitation)	Individual	(90) No	48 (720 min)	General rehabilitation	Balance (BBS)
	MB: 30	MB: 59.7 (7.6)		MB: 13/17							
	CG: 30	CG: 60.3 (8.4)		CG: 10/20							
Liu et al. [41], China	48 (0%)				30 min × unclear, 12 wks (personalized Tai Chi)	Simultaneous (General rehabilitation)	Individual	NR	NR	General rehabilitation	Balance (BBS)
	MB: 24	MB: 52.13 (14.13)	MB: 17.65 (5.34) d	MB: 9/15							
	CG: 24	CG: 53.51 (12.63)	CG: 18.73 (8.78) d	CG: 8/16							
Zhang, Li et al. [42], China	62 (0%)				40 min × 5 sessions/wk, 8 wks (Baduanjin Qigong)	Simultaneous (Usual treatment + balance training)	NR	(200 min) Yes	40 (1600 min)	Usual treatment + balance training	Balance (BBS)
	MB: 31	MB: 55.07 (4.81)	MB: 6.22 (2.45) wk	MB: 21/10							
	CG: 31	CG: 46.71 (3.57)	CG: 7.01 (1.89) wk	CG: 19/12							
Tian [43], China	60 (5%)		NR	60/0	60 min × 2 sessions/wk, 12 wks (Baduanjin Qigong)	Simultaneous (Usual treatment)	Group	(120 min) No	24 (1440 min)	Usual treatment	Balance (BBS)
	MB: 30	MB: 54.3 (4.7)									
	CG: 30	CG: 53 (4.3)									
Bai et al. [44], China	60 (0%)				40 min × 7 sessions/wk, 6 wks (Baduanjin Qigong)	Simultaneous (Balance training)	NR	(280 min) Yes	42 (1680 min)	Balance training	Balance (BBS)
	MB: 30	MB: 53.7 (4.5)	MB: 43.2 (6.53) d	MB: 18/12							
	CG: 30	CG: 51.3 (7.5)	CG: 38.5 (6.12) d	CG: 19/11							
Zhou et al. [45], China	68 (0%)	65.2 (8.5) for all participants	NR	0/68	Unclear × 2 sessions/wk, 4 wks (24-style Tai Chi)	Simultaneous (General rehabilitation)	NR	NR	8 (unclear)	Drug treatment + general rehabilitation	Balance (BBS)
	MB: 34										
	CG: 34										
Zhang, Guo et al. [46], China	224 (1.3%)	33 to 82	1 to 6 months	NR	40 min × 7 sessions/wk, 6 wks (Baduanjin Qigong)	Simultaneous (Bobath techniques + drug therapy)	NR	(280 min) Yes	24 (960 min)	Bobath techniques + drug therapy	Balance (BBS)
	MB: 115										
	CG: 106										
Li et al. [47], China	40 (0%)				40 min × 7 sessions/wk, 6 wks (Tai Chi)	Simultaneous (General rehabilitation + Prokin training)	NR	(280 min) Yes	42 (1680 min)	General rehabilitation	Balance (BBS)
	MB: 20	MB: 57.8 (7.3)	MB: 14.8 (4.6) d	MB: 13/7							
	CG: 20	CG: 58.7 (6.4)	CG: 14.5 (4.5) d	CG: 11/9							
Xu et al. [48], China	80 (0%)				40 min × 7 sessions/wk, 12 wks (Tai Chi posture)	Simultaneous (General rehabilitation)	Mixed	(280 min) Yes	84 (3360 min)	General rehabilitation	Balance (BBS)
	MB: 40	MB: 60.14 (10.25)	MB: 45.21 (25.42) d	MB: 18/22							
	CG: 40	CG: 48.23 (12.32)	CG: 47.34 (22.56) d	CG: 14/26							

Note: ISZ = initial sample size; AT = attrition rate; wk = week; M = month; yr = year; MB = mind–body exercise; CG = control group; BBS = The Berg Balance Scale; FRT = functional Reach Test; TBT = Timed Balance Test; NR = Not Reported; mode of combination indicates whether mind–body movement intervention is combined with other treatments (drug therapy or usual care). If only mind–body movement was used as the intervention program for stroke survivor, we considered “separate condition,” vice versa.

**Table 2:** Summary of methodological quality for included studies.

Author [Reference]	Item 1	Item 2	Item 3	Item 4	Item 5	Item 6	Item 7	Item 8	Item 9	Score
Taylor-Piliae et al. [27]	1	1	1	1	1	1	1	1	1	9/9
Taylor-Piliae et al. [28]	1	1	1	1	1	0	0	1	1	7/9
Schmid et al. [29]	1	1	1	0	1	0	1	1	1	7/9
Immink et al. [30]	1	1	1	1	1	0	1	1	1	8/9
Yang & Tang [31]	1	0	1	0	0	0	1	1	1	5/9
Kim et al. [32]	1	0	1	0	1	0	1	1	1	6/9
Zhou et al. [33]	1	0	1	0	1	1	1	1	1	7/9
Yang et al. [38]	1	0	1	0	1	1	1	1	1	7/9
Zhang et al. [39]	1	0	1	0	1	1	1	1	1	7/9
Fu et al. [40]	1	0	1	1	1	1	1	1	1	8/9
Liu et al. [41]	1	0	1	0	1	1	1	1	1	7/9
Zhang et al. [42]	1	0	1	0	1	1	1	1	1	7/9
Tian [43]	1	0	1	0	1	0	1	1	1	6/9
Bai et al. [44]	1	0	1	0	1	1	1	1	1	7/9
Zhou et al. [45]	1	0	1	0	1	1	1	1	1	7/9
Zhang et al. [46]	1	0	1	0	1	0	1	1	1	6/9
Li et al. [47]	1	0	1	0	1	1	1	1	0	6/9
Xu et al. [48]	1	0	1	0	1	1	1	1	1	7/9

Note: Item 1 = randomization; Item 2 = concealed allocation; Item 3 = similar baseline; Item 4 = blinding of assessors; Item 5 = more than 85% retention; Item 6 = missing data management (intention-to-treat analysis); Item 7 = between-group comparison; Item 8 = point measure and measures of variability; Item 9 = Co-intervention (should be either avoided in the trial design or similar between the index and control groups); 1 = explicitly described and present in details; 0 = absent, inadequately described, or unclear; NA = not applicable.

**Table 3:** Moderator analysis for mind–body exercises versus control group.

**Categorical Moderator**	**Level**	**No. of Studies**	**Hedge’s *g***	**95% CI**	***I*^2^, %**	**Test for between-Group Homogeneity**		
						**Q-Value**	**df (Q)**	***p*-Value**
Intervention duration	<12 weeks	13	1.64	0.92 to 2.36	92.74%	0.07	1	0.79
	≥12 weeks	6	1.47	0.41 to 2.52	96.67%			
Training frequency	<5 sessions/week	9	0.94	0.14 to 1.74	91.94%	4.69	1	0.03
	≥5 sessions/week	10	2.17	1.4 to 2.93	94.76%			
Session length	Less than 45 min	8	2.54	1.81 to 3.27	94.94%	14.21	1	0.00
	45 min or longer	10	0.66	0.01 to 1.3	81.79%			
Mode of combination	Separate	5	0.41	−1.15 to −0.80	79.98%	7.59	1	0.01
	Simultaneous	14	2.01	1.41 to 2.6	93.63%			
Weekly training dose ≥ 150-min	Yes	12	1.71	0.91 to 2.51	96.45%	0.88	1	0.35
	No	5	1.01	−0.22 to 2.24	78.18%			
Sample size	<60	8	1.23	0.26 to 2.19	87.96%	0.91	1	0.34
	≥60	11	1.84	1.03 to 2.64	96.56%			
**Continuous Moderator**	**No. of Studies**	**β**	**95% Confidence Interval**		**Q-Value**		**df**	***p***
Number of total sessions	17	0.0142	0.0039 to 0.0244		7.36		1	0.0067
Weekly training dose	17	0.00776	0.00579 to 0.00972		59.62		1	0.000

Note: mode of combination indicates whether mind–body movement intervention is combined with other treatments (drug therapy or usual care). If only mind–body movement was used as the intervention program for stroke survivor, we considered “separate condition,” vice versa.
